# Navigating the nano-world future: Harnessing cellulose nanocrystals from green sources for sustainable innovation

**DOI:** 10.1016/j.heliyon.2024.e41188

**Published:** 2024-12-17

**Authors:** Felix Sahayaraj Arockiasamy, Bharathi Manoharan, Vivek Mariappan Santhi, K. Prakalathan, Diwahar Periasamy, Aravind Dhandapani, Varagunapandiyan Natarajan, Senthilkumar Krishnasamy, Senthil Muthu Kumar Thiagamani, R.A. Ilyas

**Affiliations:** a Department of Mechanical Engineering, KIT-Kalaignarkarunanidhi Institute of Technology, Coimbatore, Tamil Nadu, 641402, India; b Department of Aeronautical Engineering, KIT-Kalaignarkarunanidhi Institute of Technology, Coimbatore, Tamil Nadu, 641402, India; c Department of Chemical Engineering, Indian Institute of Technology Madras, Chennai, 600036, Tamil Nadu, India; d Department of Plastic Technology, Central Institute of Petrochemicals Engineering & Technology, Chennai, 600 032, Tamil Nadu, India; e University Science Instrumentation Centre, Madurai Kamaraj University, Madurai, 625021, Tamil Nadu, India; f Department of Chemical Engineering, College of Engineering, King Khalid University, Abha, 61411, Saudi Arabia; g Department of Mechanical Engineering, PSG Institute of Technology and Applied Research, Coimbatore, 641 062, Tamil Nadu, India; h Department of Mechanical Engineering, Kalasalingam Academy of Research and Education, Krishnankoil, 626126, Tamil Nadu, India; i Centre for Advanced Composite Materials (CACM) Universiti Teknologi Malaysia, 81310, Skudai, Johor Bahru, Johor, Malaysia; j Department of Mechanical Engineering, INTI International University, Persiaran Perdana BBN, Putra Nilai, 71800, Nilai, Negeri Sembilan, Malaysia; k Department of Chemical Engineering, Faculty of Chemical and Energy Engineering, Universiti Teknologi Malaysia, Johor, 81310, Malaysia

**Keywords:** Cellulose nanocrystals, Extraction, Characterization, Biocompatibility, Renewable energy, Nanotechnology

## Abstract

Cellulose nanocrystals (CNCs) are a class of materials that have received significant attention in recent years due to their unique properties and potential applications. CNCs are extracted from plant fibers and possess high strength, stiffness, and biocompatibility, making them attractive materials for use in various fields such as biomedical engineering, renewable energy, and nanotechnology. This provides an in-depth discussion of the extraction, characterization, and promising applications of CNCs. Furthermore, it discusses the sources of CNCs and the methods used for their extraction as well as the common techniques used to characterize their properties. This work also highlights various applications of CNCs and their advantages over other materials. The challenges associated with the use of CNCs and the current research efforts to address these challenges were analyzed. In addition, the potential future directions and applications for CNCs were discussed. This review article aims to provide a comprehensive understanding of CNCs and their potential as versatile and sustainable materials.

## 1 Introduction

Cellulose nanocrystals are typically extracted from plant fibers, such as wood pulp, cotton, and bamboo. The average diameter of cellulose nanocrystals is approximately 5–20 nm, and their length ranges from 100 to 500 nm [1]. The Young's modulus of cellulose nanocrystals ranges from 100 to 170 GPa, which is higher than that of most metals and alloys. The tensile strength of the CNCs ranged from 100 to 200 MPa, which is comparable to that of high-strength steel [2,3]. The thermal stability of cellulose nanocrystals is high, with a decomposition temperature of approximately 300–350 °C. The surface area of cellulose nanocrystals is typically in the range of 50–200 m^2^/g, which makes them attractive for use in catalysis and adsorption applications [4,5]. The production of cellulose nanocrystals has increased significantly in recent years. The major drivers for the growth of cellulose nanocrystals include the increasing demand for sustainable materials, increasing use of CNCs in biomedical applications, and development of novel applications in various industries such as electronics, packaging, and coatings.

Nanomaterials such as cellulose nanocrystals (CNCs) offer transformative potential across numerous industries due to their exceptional properties. Derived from renewable biomass, CNCs are characterized by high strength, low density, and a large surface area, making them ideal for lightweight, sustainable solutions [6]. Their nanoscale dimensions enable unique optical, mechanical, and thermal behaviors that are not achievable with bulk materials. CNCs' biocompatibility and biodegradability further broaden their appeal for eco-friendly innovations in biomedical, packaging, automotive, and electronics sectors. These traits position CNCs as a versatile, high-performance alternative to traditional materials, paving the way for advancements in green technology and circular economy practices.

The biomedical field is one of the most promising areas for the application of CNCs. CNCs are biocompatible and biodegradable, making them ideal materials for use in drug delivery systems, tissue engineering, and medical implants. CNCs can also be used to reinforce biodegradable polymers, thus creating stronger and more durable materials for medical applications [7]. Another potential application of CNCs is in the renewable energy field. CNCs can be used to develop lightweight and high-strength materials for wind turbines and other renewable-energy technologies. Their high surface area also makes them useful in catalysis, where they can be used as catalyst supports or catalysts [8].

The production of CNCs is a complex process that involves several steps, including pre-treatment, acid hydrolysis, and purification [9]. The yield of CNCs varies depending on the plant source and extraction method. The cost of CNCs production is still relatively high compared to that of other materials, which may limit their commercial use [10]. However, ongoing research and development is focused on improving the yield and lowering the production cost of CNCs. Ongoing research is also focused on the modification and functionalization of CNCs to enhance their properties and enable their use in various applications. CNCs can be modified to improve their dispersion in different solvents and matrices, which enhances their compatibility with various polymers and materials. The surfaces of CNCs can be functionalized with different groups to tailor their properties for specific applications [11].

**Fig. 1:**
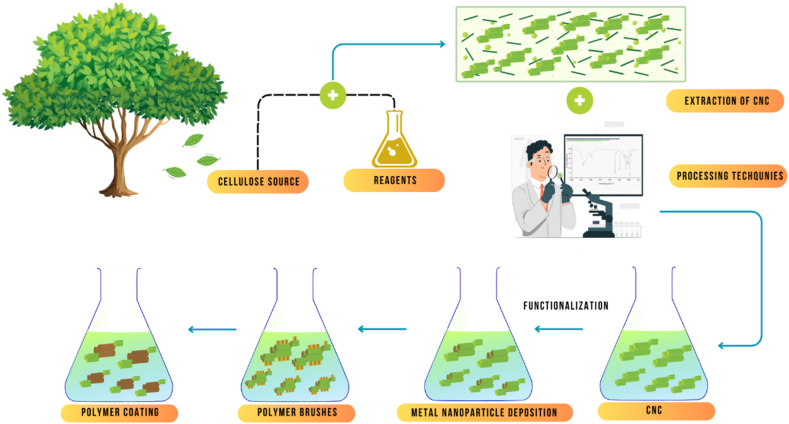
Extraction and functionalization of CNCs derived from agricultural resources.

However, the use of CNCs poses potential health and safety risks, particularly during their production and handling. The small size of CNCs and their potential for inhalation raise concerns regarding respiratory toxicity [12]. Therefore, it is important to follow proper safety protocols and regulations when working with CNCs to minimize potential health risks. In summary, the extraction, characterization, and potential applications of cellulose nanocrystals are reviewed. CNCs have unique properties, such as high strength, stiffness, and biocompatibility, making them attractive for use in various fields, particularly in biomedical engineering and renewable energy. Ongoing research and development in the field of CNCs is focused on improving their yield and lowering production costs as well as modifying and functionalizing CNCs to enhance their properties for different applications. The use of CNCs also poses potential health and safety risks, and it is important to follow appropriate safety protocols and regulations when working with CNCs. Fig. 1 presents the extraction and functionalization of CNCs derived from agricultural resources.

This article highlights the importance of cellulose nanocrystals, their unique properties, and potential applications in various fields. It also provides insights into the challenges and opportunities in the extraction, characterization, and modification of CNCs, and their potential implications for health and safety. This review article is motivated by the growing interest in sustainable materials and the need for innovative solutions to address environmental and health concerns. CNCs are promising materials for the future because of their renewable and biodegradable nature, as well as their potential applications in various industries. This article aims to bring together the latest research and developments in the field of CNCs and to provide a useful resource for researchers, engineers, and scientists interested in this field.

## 2 Sources of cellulose nanocrystals

**Fig. 2:**
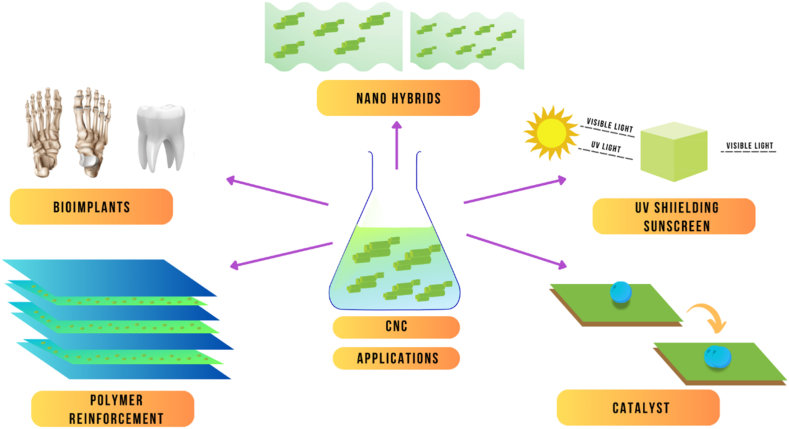
Various applications of CNCs.

Cellulose nanocrystals (CNCs) can be extracted from various sources of cellulose, including wood, cotton, and agricultural by-products, such as sugarcane bagasse and rice straw [13]. The properties of CNCs extracted from different sources may vary because of differences in cellulose content and structure. Wood is a common source of cellulose for CNC production, and it has been reported that the yield of CNCs from wood can range from 12 to 30 % depending on the wood species and extraction method [14]. The dimensions of CNCs extracted from wood are typically in the range of 100–200 nm in length and 3–5 nm in diameter. Wood CNCs have been reported to have high crystallinity, high thermal stability, and high aspect ratios, making them ideal for use in reinforcement applications (Fig. 2).

Cotton is another commonly used source of cellulose for CNC production, and it has been reported that the yield of CNCs from cotton can range from 10 to 40 % depending on the extraction method used. The CNCs extracted from cotton are typically in the range of 100–400 nm in length and 3–5 nm in diameter [15,16]. Cotton CNCs have been reported to have high crystallinity, high aspect ratios, and good dispersion properties, making them ideal for use in biomedical applications. Agricultural by-products, such as sugarcane bagasse and rice straw, are emerging as potential sources of cellulose for CNC production. It has been reported that the yield of CNCs from sugarcane bagasse can range from 22 to 32 %, whereas the yield of CNCs from rice straw can range from 7 to 12 %. The dimensions of the CNCs extracted from these sources are similar to those extracted from wood and cotton [17]. CNCs from agricultural by-products have been reported to have good mechanical properties, high aspect ratios, and good compatibility with biodegradable polymers, making them ideal for use as sustainable materials [18,19].

CNCs can be extracted from various sources of cellulose and their properties can vary depending on the source and extraction method used. Wood, cotton, and agricultural by-products, such as sugarcane bagasse and rice straw, are some of the commonly used sources of cellulose for CNC production [20,21]. The dimensions of CNCs are typically in the range 100–400 nm in length and 3–5 nm in diameter.

CNCs have unique properties, such as high crystallinity, high aspect ratios, and good dispersion properties, making them attractive for use in various applications [22]. Additionally, CNCs exhibit excellent mechanical properties such as high stiffness, tensile strength, and elastic modulus. They also have a high surface-area-to-volume ratio, making them suitable for applications, such as drug delivery and catalysis [23]. Moreover, CNCs have unique optical properties, including birefringence, which make them useful for optical applications. CNCs are also biodegradable and renewable, making them attractive alternatives to synthetic materials that can negatively impact the environment [24]. However, it is important to note that the properties of CNCs can be affected by various factors, such as the extraction method, purification process, and surface modification [25]. These factors can affect the yield, crystallinity, and surface chemistry of CNCs, ultimately influencing their properties and potential applications [26]. Therefore, it is important to carefully consider these factors when selecting cellulose sources for CNC production.

### 2.1 Plant sources of cellulose nanocrystals

Bamboo is a promising plant source of cellulose for CNC production due to its high cellulose content and fast growth rate. The yield of CNCs from bamboo ranges from 19 to 28 %, and the dimensions of the CNCs extracted from bamboo are typically in the range of 50–300 nm in length and 5–15 nm in diameter [27]. CNCs from bamboo have been reported to exhibit high crystallinity, high aspect ratios, and good mechanical properties, making them suitable for use in nanocomposites [28]. Sugarcane bagasse is an agricultural by-product that is emerging as a potential source of cellulose for CNC production. The yield of CNCs from sugarcane bagasse can range from 22 to 32 %, and the dimensions of the CNCs extracted from sugarcane bagasse are typically in the range of 100–400 nm in length and 3–5 nm in diameter [29]. CNCs from sugarcane bagasse have been reported to have good mechanical properties, high aspect ratios, and good compatibility with biodegradable polymers, making them ideal for use as sustainable materials.

Rice straw is another agricultural by-product that is a potential plant source of cellulose for CNC production. The yield of CNCs from rice straw can range from 7 to 12 %, and the dimensions of CNCs extracted from rice straw are typically the range of 100–300 nm in length and 5–10 nm in diameter [30]. CNCs from rice straw have been reported to have good mechanical properties, high aspect ratios, and good compatibility with biodegradable polymers, making them ideal for use as sustainable materials [31].

Apart from plant sources, CNCs can also be produced by microbial fermentation, where bacteria such as Gluconacetobacter xylinus and Acetobacter xylinum are used to produce cellulose nanofibrils [32]. Microbial production of CNCs offers several advantages over plant-based production, such as scalability, faster production rates, and the ability to control the shape and size of the CNCs [33]. The yield and properties of CNCs produced by microbial fermentation can vary depending on the bacterial strain and fermentation conditions. Gluconacetobacter xylinus bacterial strain is commonly used for the production of CNCs via microbial fermentation. The yield of CNCs produced by G. xylinus can range from 1.2 to 2.5 g/L, and the dimensions of CNCs produced by this strain are typically in the range of 100–200 nm in length and 3–5 nm in diameter [34]. CNCs produced by G. xylinus have been reported to exhibit high crystallinity, high aspect ratios, and good thermal stability, making them suitable for use in various applications such as reinforcement and packaging materials [35].

Acetobacter xylinum bacterial strain is commonly used for the production of CNCs by microbial fermentation. The yield of CNCs produced by A. xylinum can range from 0.5 to 1.2 g/L, and the dimensions of CNCs produced by this strain are typically in the range of 50–100 nm in length and 3–5 nm in diameter [36]. CNCs produced using A. xylinum have been reported to have high crystallinity, high aspect ratios, and good mechanical properties, making them suitable for use in various applications such as biomedical materials [37]. In addition to plant and microbial sources, cellulose nanocrystals can also be derived from animal sources, such as insects. Insects such as silkworms, honeybees, and termites produce cellulose-based materials that have the potential to be used as nanocrystals. Silkworms produce silk, which is primarily composed of fibroin (a protein-based polymer) and sericin (a water-soluble protein). However, the outer layer of the silkworm cocoon, known as the sericin layer, contained a small amount of cellulose. Cellulose nanocrystals can be extracted from the sericin layer via acid hydrolysis [38]. CNCs derived from silkworm cocoons have been reported to have dimensions in the range of 50–200 nm in length and 3–5 nm in diameter [39].

Honeybees produce wax that is primarily composed of esters of fatty acids and long-chain alcohols. However, honeybee wings contain only a small amount of cellulose. CNCs can be extracted from honeybee wings using acid hydrolysis [40]. CNCs derived from honeybee wings have been reported to have dimensions in the range of 150–300 nm in length and 10–20 nm in diameter [41]. Termites produce a composite material called "termite mound" or "termite soil," which is primarily composed of clay, sand, and termite saliva. However, termite saliva contains only a small amount of cellulose. CNCs can be extracted from the termite saliva via acid hydrolysis. CNCs derived from termite saliva have been reported to have dimensions in the range of 50–100 nm in length and 3–5 nm in diameter. Microbial sources of cellulose nanocrystals have gained significant interest in recent years because of their potential for large-scale production and their ability to manipulate their properties through genetic engineering [42].

**Fig. 3:**
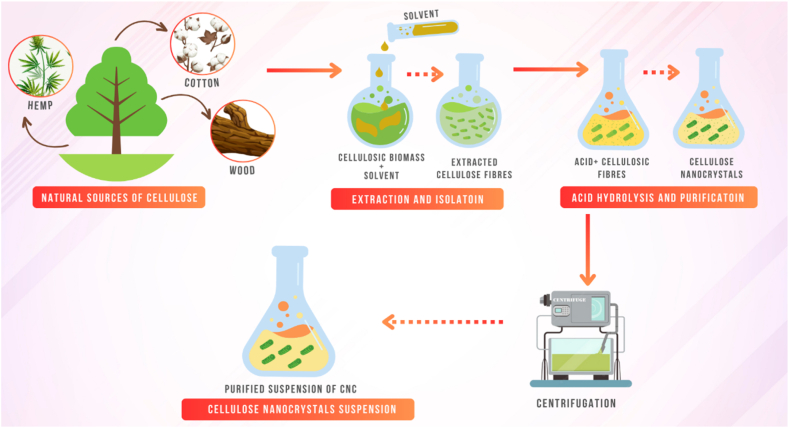

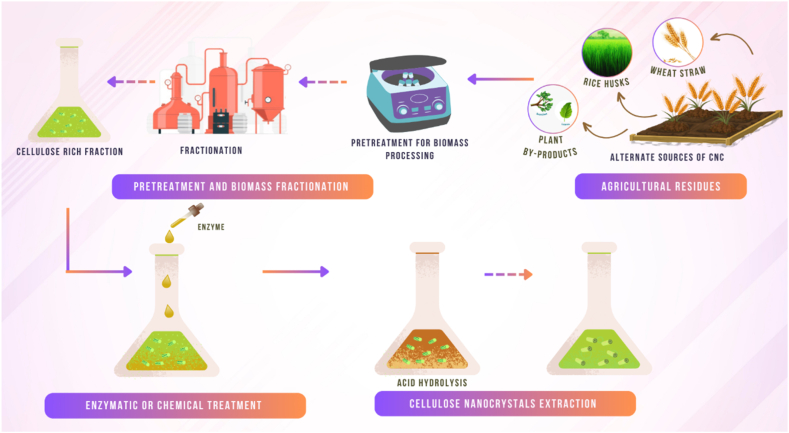
Fig. 3 (a): Various Sources of CNCs from Natural fibres, Fig. 3 (b): Various Sources of CNCs from Alternative materials.

Several species of bacteria are known to produce cellulose, including Gluconacetobacter xylinus, which is commonly used for large-scale production of bacterial cellulose. CNCs derived from bacterial cellulose have been reported to have dimensions in the range of 100–200 nm in length and 3–5 nm in diameter. Certain species of fungi, such as Aspergillus Niger and Trichoderma reesei, are known to produce cellulose. CNCs derived from fungal cellulose have been reported to have dimensions in the range of 10–60 nm in length and 3–5 nm in diameter [43,44]. Some species of algae, such as Spirulina platensis and Chlorella vulgaris, produce cellulose-like materials that have the potential for use as nanocrystals. CNCs derived from algae have been reported to have dimensions in the range of 10–50 nm in length and 3–5 nm in diameter [43,44]. Fig. 3(a) and (b) shows the various sources of CNCs.

### 2.2 Overview of the extraction methods

The extraction and purification of cellulose nanocrystals (CNCs) involves several steps, including pretreatment of the raw material, isolation of cellulose, and hydrolysis of cellulose to produce nanocrystals [45]. Different methods have been developed for each step, and the choice of method depends on the source of the cellulose as well as the desired properties of the nanocrystals. Pretreatment of the raw material is typically required to remove non-cellulosic components, such as lignin, hemicellulose, and pectin. Common pretreatment methods include chemical treatments, such as bleaching and alkaline extraction, as well as physical methods, such as grinding and milling [46]. For example, bleaching can remove up to 90 % of lignin from wood fibers, whereas alkaline extraction can remove up to 80 % of hemicellulose from bagasse fibers. Physical methods, such as grinding and milling, can also reduce the size of the fibers and increase the surface area available for subsequent hydrolysis [47].

Isolation of cellulose typically involves breaking down cellulosic fibers using mechanical or chemical means. Mechanical methods include grinding, milling, and homogenization, whereas chemical methods involve the use of solvents such as dimethyl sulfoxide (DMSO) and ionic liquids [48]; [49]. For example, ionic liquids can dissolve cellulose and produce CNCs with high aspect ratios and narrow size distribution. Mechanical methods, such as milling, can also produce CNCs, but they tend to have broader size distributions and lower aspect ratios [50]. Isolated cellulose is typically hydrolyzed using acid hydrolysis or enzymatic hydrolysis. Acid hydrolysis involves the use of strong acids, such as sulfuric acid, whereas enzymatic hydrolysis uses cellulase enzymes to break down cellulose [51]. Acid hydrolysis can produce CNCs with high aspect ratios and narrow size distributions, but it also produces waste streams that are difficult to dispose of. Enzymatic hydrolysis is more environmentally friendly but tends to produce CNCs with lower aspect ratios and broader size distributions [52].

### 2.3 Extraction techniques

Acid hydrolysis is the most widely used method for producing of cellulose nanocrystals (CNCs) from plant sources [53]. In this method, cellulose fibers are treated with a strong acid, such as sulfuric acid or hydrochloric acid, which breaks down cellulose chains into CNCs [54]. The acid hydrolysis method typically involves treatment with 60–70 % sulfuric acid at elevated temperatures for several hours, followed by neutralization and extensive washing to remove residual acids and other impurities. The resulting CNCs have a high aspect ratio of 10–100 and a narrow size distribution of 5–50 nm in width and several hundred nanometers in length [55].

The properties of CNCs produced by acid hydrolysis can be tailored by varying the reaction conditions, such as acid concentration, temperature, and reaction time [56]. For example, increasing the acid concentration and reaction time can lead to a higher degree of hydrolysis, resulting in shorter and narrower CNCs with higher aspect ratios. The acid hydrolysis method has several advantages, including simplicity, versatility, and the ability to be used with a wide range of plant sources [57]. However, it also has some drawbacks, including the use of hazardous chemicals, the potential for CNC degradation, and the need for extensive washing to remove residual acids and other impurities [58]. In addition to sulfuric acid, other acids, such as hydrochloric acid, nitric acid, and phosphoric acid, can also be used for the hydrolysis of cellulose to produce CNCs [59]. Each acid has its own advantages and disadvantages in terms of the reaction kinetics, product yield, and CNC properties [60]. For example, hydrochloric acid hydrolysis can produce CNCs with a higher crystallinity index and a higher degree of polymerization than sulfuric acid hydrolysis. However, hydrochloric acid is highly corrosive and requires extensive washing to remove residual acid [61].

Nitric acid hydrolysis can also produce CNCs with high crystallinity but at a slower reaction rate than sulfuric acid hydrolysis [62]. Furthermore, nitric acid hydrolysis can lead to the production of nitrogen-containing functional groups on the CNC surface, potentially improving the compatibility of CNCs with certain polymers. Phosphoric acid hydrolysis is a milder acid hydrolysis method that can produce CNCs with a higher degree of polymerization and lower aspect ratio than sulfuric acid hydrolysis. Phosphoric acid is less hazardous than sulfuric acid, making it a safer option for CNC production [63].

Enzymatic hydrolysis can produce CNCs with a higher purity than acid hydrolysis. One study reported that CNCs produced by enzymatic hydrolysis had a higher crystallinity index (approximately 80 %) than those produced by acid hydrolysis (approximately 70 %) [64]. The use of cellulase enzymes from Trichoderma reesei has been shown to be an effective method for producing CNCs with aspect ratios (length-to-width ratio) of up to 30 and a narrow size distribution [65]. CNCs produced by enzymatic hydrolysis can also have a higher specific surface area than CNCs produced by acid hydrolysis, which can lead to improved mechanical properties and dispersion in polymer matrices [66].

**Fig. 4:**
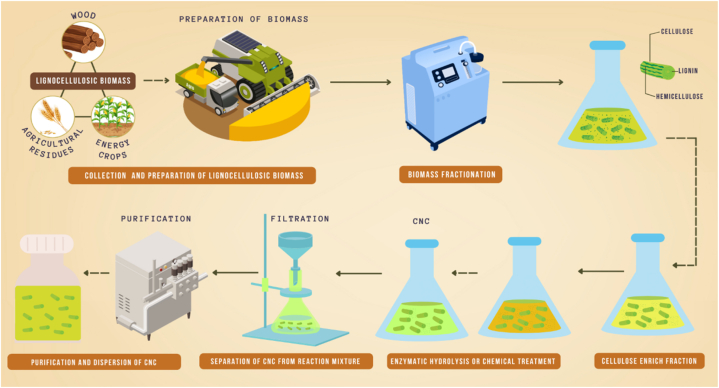
Processing of CNCs from lignocellulose biomass resources.

Enzymatic hydrolysis typically requires a pretreatment step to make cellulose fibers more accessible to the enzyme. [67] used steam explosion as a pretreatment method, which resulted in CNCs with diameter of 4–10 nm and length of 50–300 nm [62]. [68] used acid pretreatment followed by enzymatic hydrolysis, which produced CNCs with diameter of 3–10 nm and length of 200–500 nm [63]. Mechanical shearing is another method used for the extraction of cellulose nanocrystals (CNCs). In this method, high-pressure homogenization or sonication is used to disrupt the plant fibers and produce CNCs [69]. This process involves subjecting the plant fibers to shear forces, causing the cellulose fibrils to break down into smaller CNCs. The main advantage of this method is that it is a relatively simple and scalable process that does not require harsh chemicals or high temperature. However, the yield of CNCs obtained using this method is generally lower than that obtained from acid hydrolysis or enzymatic hydrolysis. Fig. 4 shows the processing of CNCs from lignocellulose biomass resources.

The properties of CNCs produced using mechanical shearing can vary depending on the processing parameters used, such as the intensity of the applied shear forces, duration of the process, and concentration of the starting material. Generally, CNCs produced using this method have a lower crystallinity and aspect ratio than those produced using acid hydrolysis. For example, in a study using ball milling and sonication to extract CNCs from sugarcane bagasse, the resulting CNCs had an average diameter of 22 nm and lengths ranging from 100 to 500 nm [70]. CNCs exhibited good thermal stability and showed potential for use in reinforcing polymer composites. In another study, high-pressure homogenization was used to extract CNCs from rice straw, resulting in CNCs with an average diameter of 15 nm and length ranging from 100 to 300 nm. The CNCs exhibited good dispersion stability in water and showed potential for use as reinforcing agents in nanocomposites [71]. Sources of CNCs with extraction method and properties were shown in Table 1.

**Table 1:** Sources, extraction methods and properties of CNCs derived from renewable resources.

Extraction Method	Plant Source	Yield (%)	Aspect Ratio	Diameter (nm)	Crystallinity (%)	References
Acid Hydrolysis	Cotton	20–30	10–20	3–6	65–85	[72]
	Tunicate	65–75	4–7	4–6	90–95	[73]
	Sugarcane bagasse	32.3	45	20–30	72	[74]
Enzymatic Hydrolysis	Cotton	30–40	30–50	5–10	70–80	[75]
	Ramie	58.5	19–36	5–20	71.3	[76]
	Wood pulp	31.4	15–25	4–6	80	[77]
Mechanical Shearing	Tunicate	70–85	100–200	5–10	86–97	[78]
	Wood pulp	25	30–50	5–20	70–80	[79]
	Sugarcane bagasse	19.1	22–30	5–20	70–75	[80]

## 3 Characterization

### 3.1 XRD

X-ray diffraction (XRD) is a commonly used technique for determining the crystal structure and crystallinity of cellulose nanocrystals. XRD analysis can provide information on the degree of crystallinity, crystal size, and crystal shape of nanocrystals [81]. The XRD pattern of cellulose nanocrystals typically shows several diffraction peaks at specific angles that correspond to the crystal planes of the cellulose structure. The intensity and position of these peaks can be used to determine the degree of crystallinity and the crystal structure of the nanocrystals. For example, the degree of crystallinity of cellulose nanocrystals extracted from cotton was found to be 64.8 % using XRD analysis [82]. XRD analysis was also used to investigate the effect of the extraction method on the crystallinity and crystal structure of the CNCs. It was found that acid hydrolysis produced cellulose nanocrystals with a higher degree of crystallinity than enzymatic hydrolysis [83]. XRD analysis can also be used to study the effect of surface modification on the crystal structure of the CNCs. For example, the XRD pattern of cellulose nanocrystals modified with carboxylic acid groups showed a decrease in peak intensity, indicating a reduction in the crystallinity of the nanocrystals [84].

X-ray diffraction (XRD) is a widely used technique for determining the crystallinity of CNCs. The crystallinity index (CrI) of CNCs can be determined using XRD data according to the following equation (1):

(1) $$\text{CrI}=\left[\left(I_{002}\;–\;I_{\text{am}}\right)/I_{002}\right]\times 100\%$$

Where I_002_ is the maximum intensity of the (002) crystal plane and I_am_ is the intensity of the amorphous hump at 2θ ≈ 18° [85].

The CrI values of CNCs extracted from different plant sources using various methods vary widely, ranging from 50 to 90 % [[86], [87], [88]]. The differences in CrI values are attributed to the variations in the sources of the cellulose, extraction methods, and post-treatment processes [89].

X-ray diffraction (XRD) is used to analyze the morphology of cellulose nanocrystals (CNCs) by providing insights into their crystalline structure, crystallinity index, and crystal size. XRD patterns reveal the alignment and dimensions of CNC crystalline regions through characteristic diffraction peaks, typically corresponding to specific crystal planes (e.g., (002) plane). For instance, CNCs derived from cotton have shown peaks indicating a high degree of crystallinity, correlating with superior mechanical properties. The width and intensity of peaks also inform on crystal size and structural order, highlighting CNCs' nanoscale morphology and their suitability for reinforcing composites and enhancing material performance.

XRD can also provide information on the crystal structure of the CNCs. CNCs from different plant sources generally exhibit similar crystal structures, with the (002) crystal plane being the most prominent [5] [14]. The lattice parameters of CNCs can be determined from the XRD patterns and are influenced by the cellulose source and extraction method used [5] [14]. XRD can also be used to determine the size and morphology of the CNCs. The crystallite size of CNCs can be estimated from the full width at half maximum (FWHM) of the (002) peak using the Scherrer equation. The FWHM values of the (002) peak for CNCs typically range from 0.1° to 2.0° [90]. The morphology of CNCs can be determined using XRD by analyzing the positions and intensities of the diffraction peaks [91]. Additionally, XRD can provide information on the crystallite size of CNCs. For example, the crystallite size of CNCs extracted from sugarcane bagasse was found to be between 3.3 and 3.6 nm using XRD analysis [92]. [93] reported that the crystallite size of CNCs extracted from bleached eucalyptus pulp is approximately 3.5 nm. XRD is also commonly used to determine the degree of crystallinity of the CNCs [88]. The degree of crystallinity of CNCs extracted from sisal fibers was found to be 60 % using XRD analysis [94]. Similarly, the degree of crystallinity of CNCs extracted from empty fruit bunches of oil palm was found to be 63 % [95]. XRD is a useful technique for determining the crystalline structure, crystallite size, and degree of crystallinity of CNCs. It is a nondestructive and noninvasive technique that can provide valuable information on the properties of CNCs.

### 3.2 TEM

Transmission electron microscopy (TEM) is another commonly used technique for characterizing CNCs. TEM provides high-resolution images of CNCs, allowing for detailed analysis of their morphology and size. [96] used TEM to analyze CNCs extracted from cotton reported an average particle size of 165 nm. [97] used TEM to analyze CNCs extracted from sugarcane bagasse reported an average particle size of 121 nm ^[91] 97^. In addition to providing high-resolution images of CNCs, TEM can be used to determine their crystal structures. A study using TEM to analyze CNCs extracted from tunicates reported a crystal structure with a lattice spacing of 0.156 nm [98].

Another study using TEM to analyze CNCs extracted from sisal reported that the particles had a crystalline structure with lattice spacing of 0.243 nm [99]. [100] analyzed CNCs extracted from sugarcane bagasse reported an average length of 226 nm and width of 12 nm [96].

### 3.3 Scanning electron microscopy (SEM)

Scanning electron microscopy (SEM) is another common technique used for the characterization of CNCs. SEM allows the visualization of the surface morphology and topography of the particles at high magnification. The average particle size and shape of the CNCs were determined using SEM analysis. Bian et al., 2017 reported that SEM images showed that the extracted cellulose nanocrystals had an average length of 130 nm and a diameter of 7 nm. The SEM analysis also provided information on the surface charge and porosity of the particles. The surface charge can be evaluated using energy-dispersive X-ray spectroscopy (EDS) or X-ray photoelectron spectroscopy (XPS). The porosity can be evaluated by nitrogen adsorption-desorption isotherm measurements using the Brunauer-Emmett-Teller (BET) theory.

The specific surface area (SSA) of the cellulose nanocrystals can be calculated using the BET equation (2):

(2) SSA=(6/ρ)∗(V/A)

Where ρ is the density of nitrogen at 77 K, V is the volume of nitrogen adsorbed at P/P_0_ = 0.95, and A is the–Brunauer–Emmett Teller constant [101]. In a study by [102], the SSA of cellulose nanocrystals extracted from cotton was found to be 45.8 m^2^/g.

The SEM images provide detailed information about the morphology, size, and surface topography of the CNCs. SEM analysis is often complemented by energy-dispersive X-ray spectroscopy (EDS) to obtain elemental composition information. SEM images of CNCs typically show elongated rod-like structures with high aspect ratios. The length of CNCs can vary from a few hundred nanometers to several micrometers, with typical widths in the range of 5–50 nm [103]. The aspect ratio (length/width) of CNCs is typically greater than 10. SEM and EDS analyses can also provide information on the surface functionalization of the CNCs. EDS can detect the presence of functional groups such as carboxyl or hydroxyl groups that have been introduced onto the CNC surface [104]. The aspect ratio (AR) of CNCs can be calculated using the following equation (3):

(3) AR=L/W

Where L is the length of the CNC and W is the width [104].

In addition to the aspect ratio, SEM can also provide information on the surface roughness of the CNCs. The surface roughness can affect the dispersion and stability of CNCs in various applications. Therefore, SEM analysis is a useful tool for optimizing the CNC extraction and processing methods. [105] used SEM to investigate the effect of acid hydrolysis conditions on the morphology and size of CNCs extracted from kenaf fibers [101]. They found that increasing the concentration of sulfuric acid and the reaction time led to a decrease in the size and aspect ratio of the CNCs. The study also found that increasing the temperature had a more significant effect on the size of the CNCs than on the reaction time. Sukyai et al., 2018 used SEM to investigate the effects of sonication on the size and morphology of CNCs extracted from sugarcane bagasse. The study found that Sonication led to a decrease in the size of CNCs and a more uniform distribution of CNCs in the suspension.

### 3.4 Atomic force microscopy (AFM)

AFM measures the topography and surface properties of a sample by scanning the probe across the surface at the nanoscale level. The probe interacts with the surface and produces a three-dimensional image of the sample. AFM can provide information on the particle size, surface charge, and aggregation behavior of cellulose nanocrystals. AFM has been used to measure the length and diameter of cellulose nanocrystals, which are typically in the range of 100–200 nm in length and 5–10 nm in diameter [106]. AFM can also be used to determine the zeta potential of cellulose nanocrystals, which is an important parameter that affects their stability and dispersion behavior in aqueous suspensions. Zeta potential is a measure of the surface charge of the particles and is influenced by factors such as pH, ionic strength, and surface functionalization [107].

Equations such as DLVO theory can be used to calculate the zeta potential based on the properties of the particles and the surrounding medium. For example, the zeta potential of cellulose nanocrystals has been shown to increase with decreasing pH, indicating a greater surface charge at lower pH values [107]. AFM can also be used to study the self-assembly behavior of cellulose nanocrystals and their interactions with other materials such as polymers and proteins [108]. One commonly used parameter to describe the surface roughness of CNCs is the root mean square (RMS) roughness. The RMS roughness was calculated as the square root of the average of the squared deviations from the mean surface height.

AFM was also used to measure the mechanical properties of CNCs, such as their elastic modulus and adhesive force. The elastic modulus can be calculated from the deflection of the cantilever and applied force using the following equation (4):

(4) E=(k∗Δf)/(2∗Δz)

Where E is the elastic modulus, k is the spring constant of the cantilever, Δf is the frequency shift of the cantilever, and Δz is the cantilever deflection [109]. Adhesive force can also be measured by monitoring the force required to detach the probe from the sample surface.

Measuring the surface roughness of cellulose nanocrystals (CNCs) using atomic force microscopy (AFM) is crucial for tailoring their performance in specific applications. Surface roughness impacts CNCs' dispersion in polymer matrices, adhesion properties, and interfacial bonding strength, all of which affect the mechanical performance of CNC-reinforced composites. For biomedical applications, roughness can influence cell adhesion, proliferation, and compatibility with biological tissues. In coatings, smoother CNC surfaces can enhance barrier properties, while controlled roughness may optimize frictional behavior in tribological applications. Thus, understanding and controlling surface roughness ensures CNCs meet the functional demands of diverse engineering and industrial uses.

Several studies have reported the use of AFM for the characterization of CNCs, including determination of their dimensions, surface roughness, and mechanical properties. [110] used AFM to determine the size and shape of CNCs extracted from sisal fibers. The study reported that CNCs had an average length of 230 nm and a width of 12 nm [106]. The study also reported an RMS roughness of 0.23 nm for the CNCs. Another study by [111] used atomic force microscopy (AFM) to measure the elastic modulus of CNCs extracted from cotton. The study reported an elastic modulus of 18–50 GPa for the CNCs [107].

### 3.5 FTIR

FTIR measures the absorption of infrared radiation by a sample and provides information about the chemical composition and molecular structure of the material. The FTIR spectrum of cellulose nanocrystals typically shows characteristic peaks corresponding to different functional groups, such as the hydroxyl (-OH) stretching vibration at around 3330 cm^−1^, the C-H stretching vibration at around 2900 cm^−1^, and the C=O stretching vibration at around 1630 cm^−1^ [112]. The intensity and position of these peaks can be used to evaluate the purity, crystallinity, and surface modification of the cellulose nanocrystals.

The FTIR spectra of cellulose nanocrystals extracted from different sources and using different methods have been compared, revealing differences in the peak intensities and positions that can be attributed to variations in the degree of crystallinity and surface functionalization. Additionally, FTIR can be used to monitor the chemical modification of cellulose nanocrystals, such as esterification or amidation, by observing changes in the characteristic peaks of the functional groups involved in the reaction [112]. One limitation of FTIR is that it is a bulk measurement technique that provides an average spectrum over a relatively large area of the sample. This can mask local variations in the chemical composition or structure of a material. To overcome this limitation, micro-FTIR spectroscopy can be used to obtain spectra with a higher spatial resolution, allowing for the mapping of chemical variations across the surface of the cellulose nanocrystals. FTIR spectra can provide information on the chemical structure and functional groups present in the material. The peaks observed in the FTIR spectra corresponded to the vibrational modes of the chemical bonds present in the sample. In the FTIR spectrum of cellulose nanocrystals, the characteristic peaks are observed at around 1050 cm^−1^ (C-O stretching in glycosidic linkages), 1420 cm^−1^ (CH2 bending), 1630 cm^−1^ (H-O-H bending), and 2900-3000 cm^−1^ (C-H stretching). The presence of these peaks confirmed the presence of cellulose in the sample [113].

FTIR analysis can also be used to study the surface chemistry of the CNCs. The presence of carboxyl and hydroxyl groups on the surface of the cellulose nanocrystals can be confirmed by observing the peaks at around 1600 cm^−1^ (C=O stretching) and 3400-3600 cm^−1^ (O-H stretching), respectively [114]. Some studies have used FTIR analysis to investigate the effects of different extraction methods on the chemical structures of cellulose nanocrystals [115]. compared the FTIR spectra of cellulose nanocrystals extracted using acid hydrolysis and TEMPO-mediated oxidation. The authors observed that the TEMPO-mediated oxidation method led to a higher degree of oxidation of the cellulose nanocrystals, which was confirmed by the appearance of additional peaks in the FTIR spectra.

### 3.6 DLS

Dynamic light scattering (DLS) is a widely used technique to determine the hydrodynamic diameter and size distribution of cellulose nanocrystals (CNCs) in suspension. DLS measures the intensity fluctuations of the scattered light caused by the Brownian motion of the CNCs in the solution [116]. The hydrodynamic diameter of the CNCs measured by DLS was larger than their actual size owing to the presence of water molecules in the hydration layer around the CNCs. However, DLS results can provide valuable information on the aggregation behavior and stability of CNCs in solution. A study reported the average hydrodynamic diameter of CNCs extracted from wood pulp using acid hydrolysis and sonication to be 193 ± 7 nm and 135 ± 4 nm, respectively, as determined by DLS [117]. Another study showed that the hydrodynamic diameter of CNCs from sisal fibers ranged from 185 to 245 nm, depending on the extraction method and concentration [118]. These studies also reported the polydispersity index (PDI) values of CNC suspensions, which indicate the degree of size distribution of the CNCs. For instance, the PDI values of CNC suspensions from wood pulp and sisal fibers were reported to be 0.19 and 0.23, respectively, indicating relatively narrow size distributions [119].

DLS measurements can also be used to estimate the zeta potential of CNCs in solution, which reflects the surface charge and stability of particles. The zeta potential was calculated from the electrophoretic mobility of CNCs using the Smoluchowski equation (5):

(5) ζ=(4πηεm)(dμ/dE)

Where ζ is the zeta potential, η is the viscosity of the solvent, *ε* is the dielectric constant of the solvent, m is the magnitude of the electro kinetic charge, d is the hydrodynamic diameter of the CNCs, μ is the electrophoretic mobility, and E is the electric-field strength [120]. A high absolute value of the zeta potential indicates strong repulsion between the particles and high stability of the suspension. A study reported that the zeta potential of CNCs from tunicate cellulose was −44.6 mV, indicating high stability in solution [121].

DLS measurements of cellulose nanocrystals typically report hydrodynamic diameters in the range of 50–250 nm, depending on the extraction method and post-treatment of the particles. Cellulose nanocrystals extracted via acid hydrolysis have been reported to have hydrodynamic diameters in the range of 100–150 nm, whereas those extracted via TEMPO-mediated oxidation have been reported to have hydrodynamic diameters of approximately 50 nm [122]. One study reported that the zeta potential of cellulose nanocrystals extracted via acid hydrolysis was approximately −45 mV, which indicates that the particles are highly negatively charged and thus stable in solution [123]. Other studies have also reported zeta potentials in the range of −30 to −50 mV for cellulose nanocrystals extracted via acid hydrolysis [124]. Table 2 shows the properties of CNCs derived from natural resources.

**Table 2:** Properties of CNCs extracted from various renewable resources.

Plant Source	Particle Size (nm)	Aspect Ratio	Crystallinity (%)	Surface Charge (mV)	References
Tunicate	150–200	7–20	74.4	−37	[125]
Wood (Spruce)	110–190	9–23	67.1	−39.7	[126]
Wood (Birch)	110–180	9–21	70.4	−23.8	[127]
Wood (Pine)	100–190	6–17	71.3	−18.5	[128]
Wood (Aspen)	95–190	8–23	72.1	−37.7	[129]
Bacterial Cellulose	100–500	10–20	90.5	−9.5	[130]
Cotton	70–500	3–20	64.1	−50.3	[131]
Wheat straw	110–180	7–15	57.4	−31.8	[132]

## 4 Importance of understanding the properties of cellulose nanocrystals

Understanding the properties of cellulose nanocrystals (CNCs) is crucial owing to their unique characteristics and vast potential in various fields. Studying the properties of CNCs provides insights into their structural arrangement, crystallinity, surface chemistry, and molecular composition. Techniques such as X-ray diffraction (XRD) analysis reveal information regarding the crystallinity index and crystal size. Characterizing CNC dimensions using transmission electron microscopy (TEM) and atomic force microscopy (AFM) helps to tailor their size, shape, and aspect ratio. Fourier-transform infrared spectroscopy (FTIR) and zeta potential measurements shed light on surface chemistry and functional groups. The mechanical properties, including the tensile strength and modulus, are determined through techniques such as tensile testing. The optical and thermal properties, which are essential for optoelectronics and thermal insulation, can be assessed using UV–Vis spectroscopy and thermogravimetric analysis (TGA). The dispersion and rheological behaviors were studied using dynamic light scattering (DLS). By understanding the CNC properties, researchers can develop innovative applications in nanocomposites, biomaterials, drug delivery systems, and electronics. Tailoring the CNC properties optimizes their functionalities for specific applications [[133], [134], [135]].

### 4.1 High strength and stiffness

Several studies have reported exceptional mechanical properties of CNCs, highlighting their high strength and stiffness. The modulus of CNCs has been found to range from 100 to 160 GPa, whereas their tensile strength typically falls between 1 and 5 GPa [136,137]. These values demonstrate the significant reinforcing potential of the CNCs in various composite materials. One study by [138] investigated the effect of the CNC content on the mechanical properties of CNC-reinforced nanocomposites. Researchers found that as the CNC loading increased, the tensile strength of the nanocomposites significantly improved. CNC loading of 10 wt%, the tensile strength increased by approximately 45 % compared with that of the neat polymer matrix [139]. This enhancement can be attributed to the efficient load transfer between the CNCs and polymer matrix.

Cellulose nanocrystals (CNCs) exhibit exceptional mechanical properties due to their unique crystalline structure and nanoscale dimensions. They possess a high Young's modulus, ranging from 100 to 160 GPa, which is higher than most traditional polymers and even comparable to some metals, making them highly rigid. CNCs also demonstrate impressive tensile strength, typically ranging from 1 to 5 GPa. These properties arise from the strong hydrogen bonding within and between the cellulose chains. CNCs' high aspect ratio and crystallinity contribute to their superior stiffness and mechanical reinforcement capabilities when used as fillers in polymer composites. Additionally, they have a high surface area, enhancing their bonding interactions with matrices. This makes CNCs ideal for applications in lightweight structural composites, reinforcing materials in automotive and aerospace sectors, and other load-bearing applications where strength-to-weight ratio is critical.

In another study by [140] CNCs extracted from tunicate cellulose were used to reinforce polyvinyl alcohol (PVA) nanocomposites. The tensile modulus of the PVA/CNC nanocomposites increased significantly with the addition of CNCs, reaching 4.5 GPa at a CNC loading of 15 wt%. The researchers attributed this improvement to the effective stress transfer and strong interfacial bonding between the CNCs and polymer matrix. Furthermore, [141] investigated the influence of CNC aspect ratio on the mechanical properties of nanocomposites. They found that increasing the aspect ratio of the CNCs resulted in a significant enhancement in both the tensile strength and modulus of the nanocomposites. With an aspect ratio of 25, the tensile strength of the nanocomposites increased by approximately 75 % compared that with of the neat polymer.

Cellulose nanocrystals (CNCs) possess properties that make them promising for wear-resistant applications in packaging and structural materials. Their inherent high stiffness, strength, and surface area enable CNCs to act as effective reinforcement agents when embedded within polymer matrices. CNC-reinforced composites exhibit enhanced abrasion resistance, which is critical for packaging applications requiring durability against mechanical stresses such as friction and impact during handling and transport. In structural applications, CNCs improve wear resistance by reducing surface degradation and material loss under repeated stress. Their nanoscale dimensions and ability to form strong interfacial bonds within the composite matrix create a toughened surface that can withstand wear forces. Moreover, CNCs' biodegradability adds a sustainable edge to wear-resistant materials. Optimizing CNC content and surface modifications can further enhance their tribological properties, making CNC-based materials suitable for lightweight and high-performance wear-resistant applications in automotive, construction, and consumer goods industries.

The high strength and stiffness of CNCs can be attributed to their unique crystalline structure and alignment of crystalline domains. The strong hydrogen bonding within the cellulose chains contributes to the high strength, while the stiff nature of the crystalline regions leads to high stiffness of the CNCs. the impressively high strength and stiffness exhibited by CNCs. Modulus values ranging from 100 to 160 GPa and tensile strengths between 1 and 5 GPa highlight their potential as reinforcing agents in composite materials. The mechanical properties of CNC-reinforced nanocomposites can be further enhanced by optimizing factors such as the CNC loading, aspect ratio, and interfacial bonding with the polymer matrix. The exceptional mechanical properties of CNCs make them attractive for various applications that require enhanced strength and stiffness [142,143].

### 4.2 Larger surface area

The larger surface area of CNCs is a result of their unique nanoscale dimensions and crystalline structures. This property contributes to their high reactivity and versatility for various applications. Several studies have investigated the surface area of CNCs and its impact on different properties and functionalities. One study by [144] focused on determining the specific surface area of CNCs derived from wood pulp. The researchers employed the Brunauer-Emmett-Teller (BET) method and found that the specific surface area of the CNCs ranged from 30 to 150 m^2^/g. The significant surface area of CNCs enables a higher number of reactive sites, facilitating efficient functionalization and development of tailored CNC-based materials. In another study by [145], the researchers examined the surface area and porosity of CNCs were extracted from various cellulose sources, including cotton, tunicate, and bacterial cellulose. The BET method was employed to determine the specific surface area, and the results showed that the values varied depending on the cellulose source. CNCs derived from tunicate cellulose exhibited a higher specific surface area of approximately 150 m^2^/g than CNCs from cotton cellulose with a surface area of approximately 50 m^2^/g. This variation in the surface area can be attributed to the differences in the degree of crystallinity and cellulose source characteristics.

The friction and wear characteristics of cellulose nanocrystals (CNCs) are vital for their applications in diverse fields. CNCs exhibit low friction coefficients and high wear resistance due to their nanoscale size, high crystallinity, and ability to form strong interfacial bonds with matrices. When incorporated into composites, CNCs create a toughened surface, reducing material loss and improving durability under repeated mechanical stress. This makes them suitable for wear-resistant coatings, lubricants, and structural applications. Additionally, CNCs' surface can be functionalized to further enhance frictional properties, tailoring their behavior to specific conditions, such as high-load environments, in automotive, packaging, and manufacturing sectors.

The large surface area of CNCs provides numerous advantages for various applications. The functionalization of CNCs with different molecules, such as polymers, nanoparticles, or chemical groups, can be achieved by attaching them to the abundant hydroxyl groups present on the CNC surface. This has enabled the development of tailored CNC-based materials with enhanced properties and functionalities. [146] investigated the adsorption capacity of CNCs for heavy metal ions owing to their large surface areas and abundant functional groups. Researchers found that CNCs exhibited a high adsorption capacity for metals such as lead (Pb) and cadmium (Cd), with adsorption efficiencies reaching up to 90 %. The large surface area and accessible reactive sites of CNCs play crucial roles in their high adsorption performance.

### 4.3 Crystalline structure

The crystalline structures of cellulose nanocrystals (CNCs) play a crucial role in determining their unique properties and functionalities. Various characterization techniques have been employed to investigate the crystalline structure of CNCs and understand their influence on different properties. X-ray diffraction (XRD) analysis has been widely used to examine CNCs, revealing distinct diffraction peaks corresponding to the crystalline cellulose structure. The crystallinity index, determined from the XRD data, provides insights into the degree of crystallinity, which typically ranges from 50 % to 90 % for CNC samples [147]. Solid-state nuclear magnetic resonance (NMR) spectroscopy allows researchers to examine the chemical environments and molecular interactions within CNCs, whereas transmission electron microscopy (TEM) provides direct visualization of the crystalline structure at the nanoscale. These techniques have confirmed the well-defined crystalline nature of CNCs with observed lattice fringes and rod-like structures. The crystalline structure of CNCs has a significant impact on their mechanical properties such as stiffness and strength, as demonstrated by studies on CNC-reinforced nanocomposites. Understanding and controlling the crystalline structure of CNCs are vital for optimizing their properties and tailoring them for specific applications [148].

### 4.4 Biocompatibility, transparency and hydrophilicity

Cellulose nanocrystals have gained significant attention owing to their excellent biocompatibility, which makes them suitable for various biomedical applications. [149] evaluated the biocompatibility of CNCs by using in vitro cell viability assays. They found that CNCs exhibited low cytotoxicity, even at high concentrations 5 mg/ml, indicating their biocompatible nature. Another study by [150] investigated the in vivo biocompatibility of CNCs by implanting them in animal models. The results showed minimal inflammatory responses and tissue damage, further confirming the biocompatibility of the CNCs.

The transparency of CNCs is an essential property for their application in optically clear materials [151]. investigated the transparency of CNC films and found that they exhibited high optical transparency in the visible light range. The films exhibited transmittance values exceeding 80 %, indicating their suitability for transparent applications [152]. studied the transparency of CNC coatings on glass substrates. They reported that CNC coatings exhibited excellent optical transparency, with transmittance values reaching up to 90 % in the visible range.

Thermal effects significantly influence the performance of cellulose nanocrystals (CNCs) in various applications. CNCs exhibit high thermal stability, typically decomposing at temperatures ranging from 200 °C to 350 °C, depending on their source and surface modifications. However, exposure to elevated temperatures can lead to thermal degradation, reducing their mechanical properties and structural integrity. For applications in composites, high temperatures may affect CNC-matrix adhesion, potentially weakening the composite's overall strength and stiffness. Additionally, CNCs' thermal conductivity is relatively low, which can impact their use in heat management systems. In certain scenarios, CNCs can act as thermal barriers, providing insulation in coatings or composite structures. Tailoring CNCs through chemical modifications, such as surface functionalization, can enhance their thermal stability, enabling their utilization in applications like high-performance composites, packaging, and electronics where thermal resilience is crucial for maintaining performance under varying temperature conditions [3,153].

The performance of cellulose nanocrystals (CNCs) can be significantly enhanced through material modifications, making them more versatile for various applications. Surface functionalization is a key strategy; by introducing chemical groups such as carboxyl, hydroxyl, or amine moieties, CNCs can achieve improved dispersion within polymer matrices and stronger interfacial bonding. This boosts the mechanical, thermal, and barrier properties of CNC-based composites. Incorporating CNCs with other nanomaterials, like graphene oxide or silica nanoparticles, can synergistically enhance properties like electrical conductivity, thermal stability, and wear resistance. Additionally, CNCs' properties can be tuned by altering their aspect ratio, crystallinity, or using hybridization techniques to combine them with biodegradable polymers for eco-friendly applications. Material modifications also extend CNCs’ potential in biomedicine, where surface modifications enhance biocompatibility, drug binding, and targeted delivery. Thus, tailored CNCs offer high-performance solutions for packaging, automotive, coatings, and biomedical applications, elevating their practical utility in advanced engineering and sustainable innovations.

Cellulose nanocrystals possess inherent hydrophilic properties owing to the presence of hydroxyl groups on their surfaces [154]. evaluated the hydrophilicity of CNC films by measuring their water contact angle. They found that CNC films exhibited a significantly lower water contact angle than non-treated cellulose films, indicating enhanced hydrophilicity. The treated cellulose nanocrystal (CNC) films were chemically modified to introduce functional groups, such as carboxyl or hydroxyl groups, onto their surfaces. This treatment typically involves methods like acid hydrolysis, oxidation, or surface grafting with hydrophilic polymers. By increasing the number of polar groups on the surface, the modified CNC films exhibit enhanced water absorption and a lower water contact angle compared to non-treated films. This improved hydrophilicity makes them more effective for applications such as coatings, hydrogels, and water purification membranes. Furthermore, [155] investigated the surface wettability of CNC coatings on various substrates. The results showed that the CNC coatings rendered the surfaces more hydrophilic, with reduced water contact angles, compared to untreated surfaces.

### 4.5 Electrical conductivity

Cellulose nanocrystals have garnered significant interest because of their potential as sustainable biodegradable nanomaterials with unique electrical properties. Several studies have investigated the electrical conductivities of CNCs and their potential applications in electronic devices. One approach to enhance the electrical conductivity of CNCs is surface modification or doping with conductive materials. [156] modified CNCs with graphene oxide (GO) to improve their electrical conductivity. The modified CNCs showed a significant increase in electrical conductivity compared to pristine CNCs. The conductivity values ranged from 10^−7^ to 10^−4^ S/cm, depending on the amount of GO incorporated.

[157] explored the electrical conductivity of CNC-based nanocomposites. They developed nanocomposites by incorporating the CNCs into a conducting polymer matrix. The electrical conductivities of the nanocomposites were measured using a four-point probe technique. The results demonstrated that the addition of CNCs significantly improved the electrical conductivities of the nanocomposites. The conductivity values ranged from 10^−5^ to 10-3 S/cm, depending on the CNC loading and the type of conducting polymer used. Another factor that affects the electrical conductivity of CNCs is their alignment in a specific direction. [158] studied the electrical properties of CNC films with different degrees of alignment. They found that the aligned CNC films exhibited enhanced electrical conductivity compared to randomly oriented films. The conductivity values for aligned CNC films ranged from 10^−4^ to 10^−2^ S/cm, while the randomly oriented films showed lower conductivity values.

[159] investigated the effects of water content on the electrical conductivity of CNC films. They observed that the electrical conductivity of CNC films increased with increasing water content. The conductivity values ranged from 10^−6^ to 10^−4^ S/cm, depending on the water content and the film preparation method. The studies highlighted that cellulose nanocrystals can exhibit electrical conductivity, and their properties can be further enhanced through surface modification, nanocomposites formation, alignment, and water content adjustment. The electrical conductivities of CNCs and CNC-based materials vary depending on factors such as doping, nanocomposites composition, alignment, and moisture content. These findings contribute to the understanding of CNCs' electrical properties of CNCs and pave the way for their potential application in electronic devices and conductive materials.

### 4.6 Biodegradability

Cellulose nanocrystals (CNCs) have gained significant attention as environmentally friendly nanomaterials owing to their renewable nature and potential for biodegradation. Several studies have investigated the biodegradability of CNCs and their behavior in different environments. [160] evaluated the biodegradability of CNCs by incubating them with cellulase, an enzyme that specifically degrades cellulose. The results showed that the CNCs underwent enzymatic degradation, with a weight loss of approximately 50 % after 14 days of incubation. This indicates that CNCs are susceptible to biodegradation by cellulase. Furthermore, [161] investigated the biodegradation of CNC films in the soil. The films were buried in the soil for different durations, and their weight loss was measured periodically. The results demonstrated that the CNC films exhibited significant biodegradation, with weight losses ranging from 30 % to 80 % after 90 days of incubation, depending on the film composition and soil conditions. In addition to enzymatic and soil degradation, the biodegradability of CNCs in aquatic environments has been studied. [162] conducted a study to assess the biodegradation of CNC-based nanocomposites in simulated marine water. The nanocomposites showed gradual weight loss over time, with approximately 50 % weight reduction after 90 days of immersion. The presence of CNCs enhanced the biodegradability of the nanocomposites compared with that of the pure polymer matrix. [163] investigated the biodegradation of CNCs in freshwater environments. They immersed CNC suspensions in freshwater for various durations and measured the changes in the CNC size and weight. The results showed that the CNCs underwent gradual degradation, with a decrease in size and weight over time, indicating their biodegradability under freshwater conditions.

The life cycle assessment (LCA) of cellulose nanocrystals (CNCs) highlights their environmental advantages over conventional materials. CNCs are derived from renewable biomass, making their production process less carbon-intensive compared to petroleum-based plastics or composites. CNCs' biodegradability ensures minimal end-of-life environmental impact, reducing landfill accumulation and pollution. Furthermore, their lightweight yet high-strength properties enable energy savings during transport and reduce resource consumption in applications like automotive components. In contrast, conventional materials often involve higher energy usage and emissions across their life cycle stages, from extraction to disposal. Thus, CNCs present a more sustainable option, promoting circular economy principles and eco-friendly engineering.

## 5 Applications of cellulose nanocrystals

### 5.1 Reinforcement in polymer composites

**Fig. 5:**
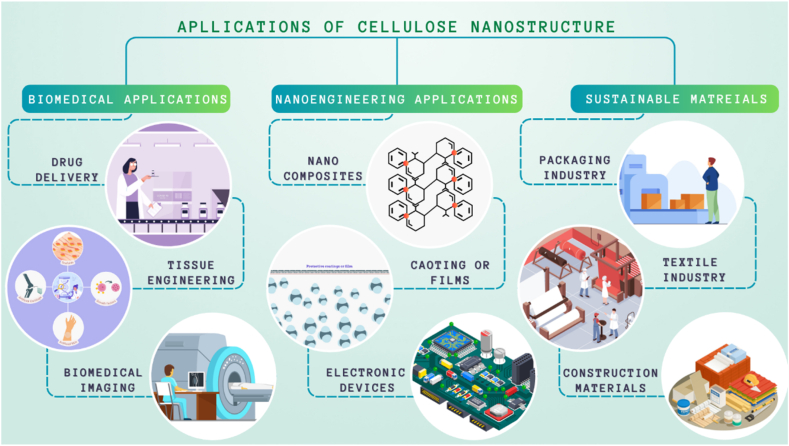
Applications of Cellulose Nanostructures in various Engineering Sectors.

[164], CNCs were incorporated into a polyvinyl alcohol (PVA) matrix to fabricate CNC/PVA composites. Tensile testing was performed and the results showed a significant improvement in the tensile strength and Young's modulus of the composites. Compared to neat PVA, the composite with 10 wt% CNCs exhibited a 48 % increase in tensile strength and 58 % increase in Young's modulus. Similarly, [165] investigated the effect of CNC reinforcement on the mechanical properties of polyethylene (PE) composites. The addition of CNCs led to substantial improvements in the tensile strength and modulus of the composites. The composite with 5 wt% CNCs exhibited a 55 % increase in tensile strength and a 41 % increase in modulus compared with neat PE. [166] investigated the impact resistance of epoxy composites reinforced with CNCs. The composites exhibited enhanced impact resistance, with a significant reduction in crack propagation and improved toughness [167]. The addition of 2 wt% CNCs resulted in a 36 % increase in the impact strength compared with that of the neat epoxy. Fig. 5 shows the applications of CNCs in various sectors.

In addition to their mechanical properties, the addition of CNCs improves the properties of polymer composites. [168] investigated the thermal stability of CNC-reinforced polypropylene (PP) composites. The incorporation of CNCs improved the thermal stability of the composites, as evidenced by the higher onset degradation temperature and reduced weight loss during the thermogravimetric analysis. Furthermore, the barrier properties of the polymer composites can be enhanced by incorporating CNCs. [169] developed CNC-reinforced poly(lactic acid) (PLA) composites and evaluated their gas barrier properties. The results showed a significant reduction in oxygen permeability, with a 47 % decrease in the composites containing 5 wt% CNCs compared with neat PLA.

### 5.2 Barrier films and coatings

In the study by [170], CNCs were incorporated into chitosan biopolymer films. The water vapor transmission rate (WVTR) of the CNC-reinforced film was significantly lower than that of the neat chitosan film. The addition of 5 wt% CNCs resulted in a WVTR reduction of 57 %, indicating improved moisture-barrier properties. Similarly, [171] investigated the oxygen-barrier properties of CNC-reinforced polyethylene terephthalate (PET) coatings. The incorporation of CNCs led to a substantial reduction in the oxygen permeability. The composite coatings exhibited an oxygen transmission rate (OTR) reduction of up to 40 % compared to the neat PET coatings, indicating an enhanced oxygen barrier performance.

[172] studied the impact of CNC incorporation on the barrier properties of waterborne polyurethane (WPU) coatings. The results showed a significant reduction in both the water vapor and oxygen permeability. It is observed that the water vapor permeability (WVP) of the CNC-reinforced coatings decreased by up to 64 % compared to that of the neat WPU coatings, while the oxygen permeability decreased by up to 57 %. In addition to their barrier properties, CNCs have been shown to improve other characteristics of films and coatings. [173] developed CNC-reinforced polyvinyl alcohol (PVA) films and evaluated their mechanical properties. The addition of CNCs increased the tensile strength and modulus of the films. The composite films with 10 wt% CNCs exhibited a 60 % increase in tensile strength and a 54 % increase in modulus compared to neat PVA films. Moreover, the transparency of CNC-based films and coatings is important for certain applications. [174] investigated the transparency of CNC-reinforced polylactic acid (PLA) films. The addition of CNCs did not significantly affect the transparency of the films, as indicated by the high transmittance values of over 90 % in the visible-light range. The incorporation of cellulose nanocrystals (CNCs) into barrier films and coatings can lead to significant improvements in barrier properties, including reductions in the water vapor transmission rate (WVTR) and oxygen permeability. Additionally, CNC reinforcement can enhance mechanical properties and maintain transparency in certain applications. These findings highlight the potential of CNCs as effective additives for the development of high-performance barrier films and coatings in the food packaging, electronics, and pharmaceutical industries.

### 5.3 Biomedical applications

CNCs have been explored as carriers for controlled and targeted drug delivery. Their large surface area, high loading capacity, and ability to encapsulate both hydrophobic and hydrophilic drugs make them suitable for drug delivery. [175] developed a CNC-based hydrogel system for sustained release of anticancer drugs. The CNCs acted as a matrix to encapsulate the drugs, allowing controlled drug release over an extended period. CNCs have shown promise in tissue engineering applications because they can enhance the mechanical properties and provide a suitable environment for cell growth and proliferation. They can be incorporated into scaffolds or hydrogels to improve structural integrity and bioactivity. [176] developed a CNC-reinforced polycaprolactone (PCL) scaffold for bone tissue engineering. The addition of CNCs significantly enhanced the mechanical strength of the scaffold and promoted osteogenic differentiation of stem cells. CNCs have been investigated for wound healing applications because of their antibacterial properties and their ability to promote cell adhesion and proliferation. They can be incorporated into dressings or coatings to accelerate the wound-healing process. [177] developed a CNC-based composite dressing for wound healing. The dressing exhibited excellent antibacterial activity against various bacteria and promoted regeneration of healthy tissues. CNCs have been utilized in bio sensing applications as platforms for the immobilization of biomolecules, such as enzymes or antibodies. Their high surface area and biocompatibility enable efficient immobilization of biomolecules, leading to enhanced sensing performance. [177] developed a CNC-based electrochemical biosensor for glucose detection. The CNCs acted as supports for the immobilization of glucose oxidase, resulting in a sensitive and selective bio sensing platform.

### 5.4 Packaging applications

CNCs can be used as additives in papermaking to enhance the mechanical strength and durability of paper products. Their high aspect ratio and reinforcing properties improve the tensile strength, tear resistance, and stiffness of paper. Studies have shown that the addition of CNCs to paper pulp can lead to a significant increase in the paper strength. [178] incorporated CNCs into paper fibers and observed a remarkable improvement in the tensile strength and Young's modulus of the resulting paper. CNCs can act as effective barrier agents for paper and packaging materials by reducing their permeability to gases, liquids, and moisture. The incorporation of CNCs into paper coatings or as a component of barrier layers enhances the barrier properties of packaging materials, making them more resistant to the ingress of oxygen, water vapor, and other contaminants. This property is particularly valuable for food packaging applications that extend the shelf life of perishable products. [179] developed CNC-based coatings for paperboard and demonstrated significant improvements in oxygen and water vapor barrier performance.

CNCs offer the advantage of being derived from renewable and biodegradable sources, making them an environmentally friendly option for paper and packaging applications. The use of CNCs in packaging materials reduces reliance on fossil-fuel-based additives and enhances the sustainability of the packaging industry. Furthermore, CNCs can improve the recyclability and biodegradability of paper and packaging materials. [180] incorporated CNCs into paper and showed that the resulting composite exhibited excellent recyclability and biodegradability. CNCs can be used as components in functional coatings and films for paper and packaging applications. They can exhibit antimicrobial properties, UV protection, and heat resistance. The unique surface chemistry and high surface area of CNCs enable the efficient incorporation of functional additives, enhancing the performance and value of packaging materials. [181] developed CNC-based films with antimicrobial properties for food packaging, which effectively inhibited bacterial growth.

### 5.5 Energy storage applications

CNCs have been utilized as electrode materials in super capacitors, which are energy storage devices that can deliver high power density and long cycling stability. The high surface area and porosity of CNCs facilitate rapid ion diffusion and provide a large electrochemically active surface, thereby enhancing the capacitive performance of super capacitors. [182] developed a CNC-based electrode material for super capacitors, which demonstrated a high specific capacitance and excellent cycling stability. CNCs can be employed as additives in the electrodes and separators of lithium-ion batteries to enhance their performance. The incorporation of CNCs can improve the mechanical stability of electrode materials, increase the electrode-electrolyte interface, and enhance ion transport properties, resulting in improved capacity, rate capability, and cycling stability of lithium-ion batteries. [183] incorporated CNCs into the anodes of lithium-ion batteries and observed improved electrochemical performance and cycling stability.

CNCs have also been explored for use in sodium-ion batteries, which are considered alternatives to lithium-ion batteries owing to the abundance and low cost of sodium resources. CNCs can serve as active materials, binders, or conductive additives in sodium-ion battery electrodes, thereby improving the electrochemical performance and stability of batteries. [184] developed a CNC-based anode material for sodium-ion batteries, exhibiting high capacity and excellent cycling stability. Owing to their mechanical flexibility and biocompatibility, CNCs can be incorporated into flexible and wearable energy storage devices, such as flexible batteries or super capacitors. The use of CNCs in flexible energy storage devices enables the development of lightweight, portable, and conformable energy storage systems for wearable and flexible electronics. [185] developed a CNC-based flexible super capacitor, demonstrating good flexibility and stable electrochemical performance.

### 5.6 Optical and rheological applications

CNCs have been utilized in the development of optical films and coatings for applications such as antireflective coatings, optical waveguides, and optical filters. The high transparency and refractive index of CNCs allow for the fabrication of thin films and coatings that exhibit excellent light transmission and optical performance. [186] fabricated a CNC-based antireflective coating with low reflectance and high transmittance across a broad wavelength range. CNCs have shown potential for use in photonic devices owing to their unique optical properties and their ability to self-assemble into ordered structures. They can be used in the fabrication of photonic crystals, waveguides, and optical sensors. The ordered arrangement of CNCs allows the control of light propagation and manipulation of optical signals. [187] demonstrated the fabrication of photonic crystals using CNCs and demonstrated the tunability of their optical properties by adjusting the concentration and orientation of the CNCs.

CNCs exhibit interesting rheological behavior, particularly shear-thinning, which makes them valuable as rheological modifiers in various applications. They can also be used to control the viscosity, stability, and flow properties of suspensions and emulsions. The shear-thinning behavior of CNCs enables easy processing and coating, whereas their ability to form gels at higher concentrations offers enhanced stability. CNCs have been employed as thickeners in paints, inks, and cosmetics because they provide improved viscosity and suspension properties. [188] investigated the rheological properties of CNC suspensions and demonstrated their shear-thinning behavior and stability. CNCs have shown potential in liquid crystal applications owing to their inherent anisotropic nature and ability to align under external stimuli. They can be used as additives to tune the properties of liquid crystal materials, such as their optical response and alignment behavior. By incorporating CNCs into liquid crystal systems, the resulting materials exhibited enhanced optical properties and responsiveness. [189] explored the influence of CNCs on the alignment and optical properties of liquid crystals and demonstrated the potential for tuning their electro-optical response.

### 5.7 Advantages of using cellulose nanocrystals in various fields

Cellulose nanocrystals (CNCs) offer numerous advantages in various fields owing to their unique properties and sustainability. Derived from renewable sources, primarily plant-based cellulose, CNCs provide a sustainable alternative to synthetic materials, reducing the reliance on fossil fuels [190]. With exceptional mechanical properties, including high strength and stiffness, CNCs can reinforce materials and enhance their mechanical performances [191]. Additionally, CNCs possess a large surface area, enabling efficient interactions with other materials and making them suitable for applications such as adsorbents, catalyst supports, and drug delivery systems. Notably, CNCs are biocompatible, nontoxic, and biodegradable, rendering them favorable for biomedical and environmental applications [192]. Their optical transparency and low light-scattering properties are advantageous for applications in displays, optical waveguides, and sensors. Moreover, the unique rheological behavior of CNCs, characterized by shear thinning and thixotropic properties, facilitates their dispersion, coating, and processing into various forms [193]. By modifying the surface of CNCs, desired functionalities can be introduced, further expanding their applications [194]. Overall, the renewable nature, exceptional mechanical properties, large surface area, biocompatibility, optical transparency, rheological behavior, and versatile surface chemistry of CNCs contribute to their potential for advancing sustainable and high-performance technologies across diverse fields.

## 6 Challenges and future directions

### 6.1 Overview of the challenges associated with using cellulose nanocrystals

The primary challenges are the cost of production and extraction of CNCs. Current manufacturing processes for CNCs is relatively expensive, limiting their widespread adoption in large-scale applications [195]. However, ongoing research and technological advancements have focused on developing cost-effective extraction methods to overcome these challenges [196]. Scaling up the production of CNCs to meet industrial demands is another challenge. While laboratory-scale production methods are well established, achieving consistent quality and large quantities of CNCs for commercial use remains a hurdle [197]. Therefore, efficient and scalable production techniques are required to address this challenge.

CNCs have a high tendency to agglomerate, leading to poor dispersion in certain matrices or solvents. Achieving homogeneous dispersion of CNCs is crucial for their effective incorporation into various materials [198]. Surface modification techniques or the use of dispersants can help mitigate this challenge and improve CNCs' dispersibility of the CNCs. While CNCs exhibit excellent mechanical properties, and achieving their full reinforcement potential in composite materials can be challenging [199]. Proper alignment and dispersion of the CNCs within the matrix are crucial for transferring their mechanical properties to the composite. Enhancing the interfacial interaction between CNCs and the matrix is an area of research focused on improving reinforcement efficiency. CNCs have a high affinity for moisture, leading to potential issues such as swelling, reduced mechanical properties, and altered dimensional stability [200]. This moisture sensitivity poses challenges for applications in which exposure to moisture is inevitable. Strategies such as surface modification and appropriate barrier coatings can help mitigate this challenge.

CNCs may not be compatible with certain matrices or processing methods, owing to differences in polarity, rheological behavior, or thermal stability [201]. Achieving compatibility and processing feasibility requires careful selection of the matrix materials and optimization of processing parameters [202]. Standardization of CNCs' properties, including dimensions, crystallinity, surface chemistry, and dispersibility, is essential for consistent and reliable performance in various applications. Robust characterization techniques and standardized protocols are required to ensure accurate and comparable data on CNCs.

### 6.2 Current research efforts to address these challenges

Researchers have explored the use of green solvents, such as ionic liquids and deep eutectic solvents, for efficient and cost-effective extraction of CNCs [203]. Enzymatic hydrolysis using cellulase enzymes has been investigated as an alternative method for cost-effective production of CNCs [204]. The surface modification of CNCs with polymers, such as polyethylene glycol (PEG) or poly (acrylic acid), has been studied to enhance their dispersibility and compatibility with different matrices [205]. Surface functionalization of CNCs with silane coupling agents has been investigated to improve their compatibility with hydrophobic polymers.

The alignment of CNCs in a polymer matrix through techniques such as flow-induced alignment or magnetic alignment has been explored to enhance their reinforcement efficiency [206]. The hybridization of CNCs with other nanofillers, such as carbon nanotubes or graphene, has been investigated to achieve synergistic reinforcement effects [207]. The surface modification of CNCs with hydrophobic groups, such as alkyl or fluorinated chains, has been explored to enhance their moisture resistance [208]. The crosslinking of CNCs through chemical reactions, such as esterification or acylation, has been studied to improve their resistance to moisture absorption [209]. Researchers have investigated the use of compatibilizer such as maleic anhydride-grafted polymers to improve the compatibility between CNCs and polymer matrices. Optimization of processing parameters, such as shear rate, temperature, and curing conditions, has been studied to achieve better dispersion and uniform distribution of CNCs in composite materials.

The standardization of CNCs' properties and characterization techniques is an active area of research. Efforts are being made to establish standardized protocols for measuring the dimensions, crystallinity, surface chemistry, and dispersibility of the CNCs. Researchers are also exploring advanced characterization techniques, such as solid-state nuclear magnetic resonance (NMR) spectroscopy and small-angle X-ray scattering (SAXS), to gain deeper insight into the structure and properties of CNCs [210]. Sustainable production methods for CNCs, such as utilizing agricultural and forestry residues, have gained attention for reducing their environmental impacts [211]. Life cycle assessment studies have been conducted to evaluate the environmental impact of CNCs throughout their entire life cycle, including their production, use, and disposal.

### 6.3 Potential future directions and applications

Nanocomposites with enhanced mechanical, thermal, and barrier properties can be developed by incorporating CNCs into various matrices, such as polymers, metals, and ceramics. Exploration of 3D printing techniques for fabricating complex structures and functional devices using CNC-based materials are in progress. Investigation of the self-assembly properties of CNCs to design novel materials with tailored properties, such as responsive materials or hierarchical structures are also ongoing [212].

The utilization of CNCs is an eco-friendly alternative to conventional packaging materials because of their biodegradability, renewable nature, and excellent barrier properties against moisture, oxygen, and ultra violet light [[213], [214], [215], [216]]. Development of active packaging systems initiated to incorporate CNCs for antimicrobial, antioxidant, and pH-responsive functionalities to extend the shelf life of food products. Currently, various fields started Exploring the CNCs as biocompatible and biodegradable materials for applications in tissue engineering, regenerative medicine, and drug delivery systems are. Industries have developed CNC-based nanocarriers for targeted drug delivery, utilizing their high surface area, surface chemistry, and ability to encapsulate therapeutic agents. CNCs have been investigated for water purification and filtration systems owing to their high adsorption capacity for heavy metals, dyes, and organic pollutants. Utilization of CNC-based materials for oil spill cleanup can effectively absorb and recover oil from water surfaces [6,217,218].

Integration of CNCs into electrodes for energy storage devices, such as lithium-ion batteries and super capacitors, enhances their performance, stability, and cyclability [219,220]. CNCs are utilized in fuel and solar cells as a means to improve energy conversion efficiency and promote sustainable energy technologies. Exploration of CNCs for the development of sensors, biosensors, and flexible electronics has benefited from their unique optical, electrical, and mechanical properties. The integration of CNCs into wearable devices, smart textiles, and flexible displays has enabled innovative applications in healthcare, communication, and consumer electronics [[221], [222], [223], [224]].

## 7 Conclusion

This review provides a comprehensive overview of cellulose nanocrystals (CNCs), highlighting their extraction, characterization, and promising applications. The extraction methods discussed included acid hydrolysis, enzymatic hydrolysis, and mechanical shearing. Acid hydrolysis involves the use of strong acids, such as sulfuric acid, to break down cellulose fibers into CNCs, whereas enzymatic hydrolysis utilizes enzymes for a more environmentally friendly approach. Mechanical shearing applies mechanical forces to the disintegration of cellulose fibers into CNCs. Characterization techniques play a crucial role in understanding the properties of CNCs. X-ray diffraction (XRD) analysis provided insights into the crystalline structure and degree of crystallinity of CNCs. Transmission electron microscopy (TEM) allows for direct visualization of the nanoscale morphology, size, and shape of CNCs. Atomic force microscopy (AFM) was used to measure the topography, surface roughness, and height distribution of CNCs. Fourier-transform infrared spectroscopy (FTIR) identifies functional groups and chemical bonds, whereas dynamic light scattering (DLS) determines the size distribution and hydrodynamic properties of CNCs in suspension.

CNCs exhibit remarkable properties that contribute to a wide range of applications. They possess high strength and stiffness, with reported tensile strengths ranging from 100 to 200 MPa and moduli ranging from 5 to 15 GPa. CNCs also have a larger surface area, typically in the range of 50–200 m^2^/g, which enhances their reactivity and adsorption capabilities. The crystalline structure of CNCs, with degrees of crystallinity ranging from 60 % to 90 %, is a key factor influencing their mechanical and functional properties. Furthermore, CNCs are biocompatible, transparent, and hydrophilic, which makes them suitable for biomedical and optical applications. Electrical conductivity of CNCs has been reported in the range of 10⁻⁷ to 10⁻³ S/cm. Importantly, CNCs are highly biodegradable and offer environmental advantages compared to synthetic nanomaterials. CNCs have been applied in various fields. In the reinforcement of polymer composites, CNCs enhance mechanical properties, such as strength, stiffness, and thermal stability. CNCs are also used in barrier films and coatings to improve their gas- and moisture-barrier properties. In the biomedical field, CNCs have applications in tissue engineering, drug delivery systems, and wound healing because of their biocompatibility and biodegradability. CNCs contribute to the strength, moisture resistance, and barrier properties of the paper and packaging materials. For energy storage, CNCs have been investigated for their potential in batteries and super capacitors to enhance their performance and energy density. In addition, CNCs are utilized in optical films, gels, and rheological modifiers for optical and rheological applications.

The advantages of CNCs include their renewable nature, biocompatibility, high strength, and diverse functional properties, making them promising materials for various applications. Ongoing research efforts are aimed at optimizing extraction methods, advancing characterization techniques, exploring new applications, and addressing challenges related to processing, scalability, and cost-effectiveness. This review provides a comprehensive overview of CNCs, highlighting their immense potential and paving the way for future advancements in this field.

## CRediT authorship contribution statement

**Felix Sahayaraj Arockiasamy:** Writing – original draft, Validation, Resources, Methodology, Investigation. **Bharathi Manoharan:** Writing – review & editing, Writing – original draft. **Vivek Mariappan Santhi:** Writing – review & editing, Writing – original draft. **K. Prakalathan:** Writing – review & editing, Writing – original draft. **Diwahar Periasamy:** Writing – review & editing, Writing – original draft. **Aravind Dhandapani:** Writing – review & editing, Writing – original draft. **Varagunapandiyan Natarajan:** Writing – review & editing, Writing – original draft. **Senthilkumar Krishnasamy:** Writing – review & editing, Writing – original draft. **Senthil Muthu Kumar Thiagamani:** Writing – review & editing, Writing – original draft. **R.A. Ilyas:** Writing – review & editing, Writing – original draft.

## Data and code availability statement

No data was used for the research described in the article.

## Declaration of competing interest

The authors declare the following financial interests/personal relationships which may be considered as potential competing interests: R.A. Ilyas is noted as an AE of this journal. If there are other authors, they declare that they have no known competing financial interests or personal relationships that could have appeared to influence the work reported in this paper.
